# Epilepsy Gene Therapy Using an Engineered Potassium Channel

**DOI:** 10.1523/JNEUROSCI.1143-18.2019

**Published:** 2019-04-17

**Authors:** Albert Snowball, Elodie Chabrol, Robert C. Wykes, Tawfeeq Shekh-Ahmad, Jonathan H. Cornford, Andreas Lieb, Michael P. Hughes, Giulia Massaro, Ahad A. Rahim, Kevan S. Hashemi, Dimitri M. Kullmann, Matthew C. Walker, Stephanie Schorge

**Affiliations:** ^1^Department of Clinical and Experimental Epilepsy, UCL Queen Square Institute of Neurology, University College London, London WC1N 3BG, United Kingdom,; ^2^UCL School of Pharmacy, University College London, London WC1N 1AX, United Kingdom, and; ^3^Open Source Instruments Inc., Watertown, Massachusetts 02472

**Keywords:** EEG, epilepsy, gene therapy, lentivirus, potassium channel, seizure detection

## Abstract

Refractory focal epilepsy is a devastating disease for which there is frequently no effective treatment. Gene therapy represents a promising alternative, but treating epilepsy in this way involves irreversible changes to brain tissue, so vector design must be carefully optimized to guarantee safety without compromising efficacy. We set out to develop an epilepsy gene therapy vector optimized for clinical translation. The gene encoding the voltage-gated potassium channel Kv1.1, *KCNA1*, was codon optimized for human expression and mutated to accelerate the recovery of the channels from inactivation. For improved safety, this engineered potassium channel (EKC) gene was packaged into a nonintegrating lentiviral vector under the control of a cell type-specific *CAMK2A* promoter. In a blinded, randomized, placebo-controlled preclinical trial, the EKC lentivector robustly reduced seizure frequency in a male rat model of focal neocortical epilepsy characterized by discrete spontaneous seizures. When packaged into an adeno-associated viral vector (AAV2/9), the EKC gene was also effective at suppressing seizures in a male rat model of temporal lobe epilepsy. This demonstration of efficacy in a clinically relevant setting, combined with the improved safety conferred by cell type-specific expression and integration-deficient delivery, identify EKC gene therapy as being ready for clinical translation in the treatment of refractory focal epilepsy.

**SIGNIFICANCE STATEMENT** Pharmacoresistant epilepsy affects up to 0.3% of the population. Although epilepsy surgery can be effective, it is limited by risks to normal brain function. We have developed a gene therapy that builds on a mechanistic understanding of altered neuronal and circuit excitability in cortical epilepsy. The potassium channel gene *KCNA1* was mutated to bypass post-transcriptional editing and was packaged in a nonintegrating lentivector to reduce the risk of insertional mutagenesis. A randomized, blinded preclinical study demonstrated therapeutic effectiveness in a rodent model of focal neocortical epilepsy. Adeno-associated viral delivery of the channel to both hippocampi was also effective in a model of temporal lobe epilepsy. These results support clinical translation to address a major unmet need.

## Introduction

Epilepsy affects 40–60 million people worldwide (Ngugi et al., 2010). Even with optimal treatment, ∼30% of people remain resistant to pharmacotherapy (Kwan et al., 2011). The development of new antiepileptic drugs has had little impact on refractory epilepsy; affected individuals experience major comorbidities, social exclusion, and an annual rate of sudden unexpected death of 0.5–1% (Devinsky, 2011; Hoppe and Elger, 2011). Although surgical resection of the epileptogenic zone can result in seizure freedom, it is unsuitable for >90% of patients with refractory epilepsy (Lhatoo et al., 2003). Surgical intervention in focal neocortical epilepsy (FNE) is further complicated by the high risk of damage to eloquent regions of the cortex (i.e. areas critically involved in memory, language, sensation or motor function) (Schuele and Lüders, 2008).

Gene therapy is a promising option to treat refractory focal epilepsy (Kullmann et al., 2014), but major hurdles remain in achieving stable, predictable, and safe transgene expression. Because focal seizures often arise from brain areas close to eloquent cortex, lentiviral vectors, which lead to rapid, stable, and, most importantly, spatially restricted transgene expression (Lundberg et al., 2008), are an attractive delivery tool. In addition, their large packaging capacity allows for a wide choice of promoter–transgene combinations, which can further increase the specificity of expression. Hitherto, clinical trials with lentivectors for CNS disorders have mainly used *ex vivo* treatment of hematopoietic stem cells (Cartier et al., 2009; Biffi et al., 2013), although a recent trial in Parkinson's disease (PD) relied on a lentivector injected directly into the striatum (Palfi et al., 2014). A larger number of trials have used adeno-associated virus (AAV) vectors to treat CNS and ophthalmic disorders including PD (Muramatsu et al., 2010; LeWitt et al., 2011; Mittermeyer et al., 2012), spinal muscular atrophy (Mendell et al., 2017), Canavan disease (Leone et al., 2012), Batten disease (Worgall et al., 2008), Sanfilippo syndrome type B (Tardieu et al., 2017), Leber's congenital amaurosis (Maguire et al., 2008), and choroideremia (MacLaren et al., 2014). Although they have a smaller packaging capacity than lentivectors, AAVs also support stable transgene expression (up to 15 years in nonhuman primates; Sehara et al., 2017), and their ability to spread further through the brain parenchyma potentially makes them better suited to treat diffuse seizure foci.

We have previously shown that lentivector-mediated overexpression of the human voltage-gated potassium channel Kv1.1 (encoded by *KCNA1*) can suppress pathological high-frequency electrocorticography (ECoG) activity in a model of FNE induced by tetanus neurotoxin (TeNT) injection into the rat motor cortex (Wykes et al., 2012). However, in this model, which mimics an especially pharmacoresistant form of FNE, epilepsia partialis continua (EPC; Cockerell et al., 1996), discrete seizures lasting >5 s are rare (see also Kätzel et al., 2014), so the effectiveness of potassium channel gene therapy in more common forms of epilepsy remains to be demonstrated.

Gene therapy based on the overexpression of Kv1.1 requires effective targeting of transgene expression to excitatory neurons. The strong viral promoter CMV successfully drives *KCNA1* overexpression in rat pyramidal neurons (Wykes et al., 2012). However, recent data suggest that CMV cannot support excitatory neuron-specific expression in nonhuman primates (Yaguchi et al., 2013; Lerchner et al., 2014). Furthermore, current clinical guidance for lentiviral gene therapy seeks to reduce the risk of mutagenesis associated with integration into the genome (Hacein-Bey-Abina et al., 2003; Baum et al., 2004).

To bring potassium channel gene therapy closer to the clinic, we have designed a construct that boosts Kv1.1 expression and reduces its inactivation with an engineered potassium channel (EKC) gene, and improves safety with a cell type-specific (*CAMK2A*) promoter. The construct was packaged into both a nonintegrating lentiviral vector and an AAV2/9 vector, and was tested for efficacy in models of FNE and of the commonest type of focal epilepsy, temporal lobe epilepsy (TLE).

## Materials and Methods

### 

#### 

##### Molecular biology.

Lentiviral and AAV transfer plasmids were constructed using standard subcloning techniques. *KCNA1* was codon optimized for human expression using GeneOptimizer software, and was synthesized using GeneArt (Thermo Fisher Scientific). All plasmids were fully sequenced before use. Sequences are available on request.

##### Voltage-clamp recordings.

Neuro-2a cells were grown in Gibco DMEM plus GlutaMAX (Thermo Fisher Scientific) supplemented with 10% heat-inactivated fetal bovine serum (Thermo Fisher Scientific), 1% penicillin/streptomycin (Thermo Fisher Scientific), and 1% nonessential amino acids (Sigma-Aldrich). Cultures were maintained in logarithmic growth phase in a humidified 5% CO_2_ atmosphere at 37°C. Transfections were performed according to the manufacturer instructions using TurboFect Transfection Reagent (Thermo Fisher Scientific). Transfected cells were plated onto 13 mm borosilicate glass coverslips (VWR). Coverslips were placed into the chamber of a BX51WI fixed-stage Upright Microscope equipped with UMPLFLN 10× and LUMPLFLN 40× water-immersion objectives (Olympus). Coverslips were submerged in a static bath of extracellular solution with the following composition (in mm): 140 NaCl, 4 KCl, 1.8 CaCl_2_, 2 MgCl_2_, and 10 HEPES, pH 7.35, osmolarity ∼301 mOsm/L. Filamented borosilicate glass micropipettes (GC150-F; Warner Instruments) were pulled to tip resistances between 2.0 and 3.0 MΩ using a P-97 Flaming/Brown Micropipette Puller (Sutter Instrument). Micropipettes were filled with an intracellular solution of the following composition (in mm): 140 KCl, 10 HEPES, and 10 EGTA, pH 7.35, osmolarity ∼291 mOsm/L. Macroscopic currents were recorded under voltage clamp using the whole-cell patch-clamp configuration. The following voltage step protocol was used: cells were held at a resting potential of −80 mV, and currents were evoked by 200 ms depolarizing steps delivered in 10 mV increments up to +20 mV. A 40 ms hyperpolarizing step to −100 mV was included before returning to baseline (Bl). Data were filtered at 3 kHz and acquired at 10 kHz using WinWCP software (J. Dempster, University of Strathclyde, Glasgow, UK) and an Axon Multiclamp 700B amplifier (Molecular Devices). Series resistance compensation was used throughout, with prediction and correction components adjusted to 80% and the bandwidth set to 1.2 kHz. Cells with series resistance of >10 MΩ were excluded from the analysis. All recordings were made at room temperature (23–26°C). The liquid junction potential, calculated to be +4.1 mV, was left uncorrected. Leak currents were minimal and left unsubtracted.

For analysis, evoked currents were taken as the steady-state current in the last 40 ms of each voltage step. Baseline holding currents were subtracted before division by cell capacitance to generate current density values. To calculate normalized conductance, the current density at each voltage step was divided by the step potential minus the potassium reversal potential (−91.34 mV). This generates raw conductance values that are corrected for the variation in K^+^ driving force that accompanies stepwise changes in membrane potential. Plots of raw conductance against voltage for each EKC-transfected cell were fit with individual Boltzmann functions given by the following equation:

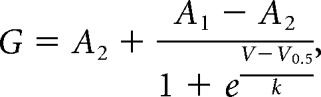
 where *G* is the conductance, *V* is the voltage, *A*_1_ is the initial (minimum) conductance, *A*_2_ is the final (maximum) conductance, *V*_0.5_ is the voltage of half-maximal conductance, and *k* is the slope factor. Raw conductance values were normalized to *A*_1_ and *A*_2_ of their own Boltzmann functions. Normalized conductance was then plotted against voltage for all EKC-transfected cells, and mean values were fitted with a single Boltzmann function.

##### Lentiviral synthesis.

The Lenti-CMV-*KCNA1* vector was identical to that used in the study by Wykes et al. (2012). For the Lenti-CaMKII-EKC vector and its Lenti-CaMKII-green fluorescent protein (GFP) control, human embryonic kidney 293T (HEK293T) producer cells were grown in Gibco DMEM + GlutaMAX (Thermo Fisher Scientific) supplemented with 10% heat-inactivated fetal bovine serum and 1% penicillin/streptomycin. Cultures were maintained in logarithmic growth phase in a humidified 5% CO_2_ atmosphere at 37°C. Cells were split every 3–4 d using 0.05% trypsin-EDTA (Thermo Fisher Scientific) and never grown for >15 passages. Cells were cotransfected with pMDG-VSV.G, pCMVdR8.74^D64V^, and either the Lenti-CaMKII-EKC or Lenti-CaMKII-GFP transfer plasmids. The mass ratio of envelope to packaging to transfer plasmids was 1:2.5:1.5. Transfections were performed according to the manufacturer instructions using Lipofectamine 2000 (Thermo Fisher Scientific). The transfection medium was replaced after 18 h. Two media harvests were collected at 40 and 60 h after transfection. Harvested media were precleaned by centrifugation at 1000 rpm for 3 min at 4°C and filtered through 0.45 μm micropores. Media were overlaid on a sucrose solution with the composition (in mm) 50 Tris-HCl, 100 NaCl, and 0.5 EDTA, pH 7.4, 10% w/v sucrose, and were centrifuged at 20,000 rpm for 2 h at 4°C. Lentiviral pellets were resuspended in sterile PBS, aliquoted, snap frozen, and stored at −80°C. Viral titer was approximated using the Lenti-X p24 Rapid Titer Kit (Clontech). Each titration was performed in triplicate with three separate aliquots. Estimated titers were 2.42 × 10^9^ infectious units (IU)/ml (Lenti-CaMKII-EKC) and 4.26 × 10^9^ IU/ml (Lenti-CaMKII-GFP).

##### AAV synthesis.

The recombinant AAV2/9 (rAAV2/9) AAV-CaMKII-EKC vector was produced in HEK293T cells grown in Gibco DMEM + GlutaMAX supplemented with 10% heat-inactivated fetal bovine serum and 1% penicillin/streptomycin, and maintained in a humidified 5% CO_2_ atmosphere at 37°C. Cells were cotransfected with the AAV2 inverted terminal repeat (ITR)-containing AAV-CaMKII-EKC transfer plasmid, a helper plasmid expressing AAV2 rep and AAV9 cap, and a third plasmid expressing the adenovirus helper functions (Streck et al., 2006). Transfections were performed using polyethylenimine MAX (Polysciences) and a plasmid ratio of 1:1:3, respectively. Cells were harvested 72 h after transfection and were lysed with three freeze–thaw cycles (−80°C to 37°C) combined with regular vortexing in lysis buffer (150 mm NaCl, 50 mm Tris-HCl, pH 8.5) and a final benzonase (Sigma-Aldrich) treatment at 37°C for 1 h. The rAAV2/9 vector was purified from the lysate using iodixanol gradient ultracentrifugation. The lysate was overlaid on increasing concentrations (15%, 25%, 40%, and 60%) of iodixanol (OptiPrep, Sigma-Aldrich) in ultracentrifuge tubes (Beckman Instruments), and centrifuged for 3 h at 2,00,000 × *g* in a SW 40 Ti rotor (Beckman Instruments). The rAAV2/9 vector was extracted from the 40% fraction with a 19 gauge needle, diluted in sterile PBS, sterilized by filtration through 0.22 μm micropores, and concentrated using Vivaspin 20 centrifugal concentrators (100,000 molecular weight cutoff; Sartorius). The final concentrated vector was stored at −80°C. Vector titer was measured via quantitative PCR using the Applied Biosystems StepOnePlus Real-Time PCR System (Thermo Fisher Scientific). Serial dilutions of the AAV-CaMKII-EKC transfer plasmid (10^1^–10^9^ plasmid copies/μl) were used as a template to create a standard curve. The reaction mixture comprised 5 μl of iTaq Universal SYBR Green Supermix (Bio-Rad), 1 μl each of the forward (5′-CAGCACGCCTTCAAGACC-3′) and reverse (5′-AAGACTTCCTCTGCCCTCAC-3′) primers at a concentration of 100 nm, 2 μl of DNA from either the plasmid standard or final vector, and 1 μl of distilled water. The PCR protocol consisted of an initial denaturation step at 95°C for 30 min, 40 cycles of denaturation at 95°C for 5 min, annealing at 58°C for 15 min, and extension at 72°C for 10 min, and a final melt curve stage. The reaction was performed in duplicate using three different dilutions of the concentrated vector sample (1:100, 1:1000, and 1:10,000 in distilled water). The estimated titer was 8.3 × 10^14^ viral genomes/ml. The rAAV2/9 AAV-CaMKII-GFP control vector was commercially synthesized by VectorBuilder using an AAV2 ITR-containing transfer plasmid designed and constructed in-house.

##### Surgical procedures.

All experiments were performed in accordance with the United Kingdom Animals (Scientific Procedures) Act 1986. For the FNE model, adult male Sprague Dawley rats (weight, 300–400 g) were anesthetized and placed into a stereotaxic frame (Kopf). Fifteen nanograms of TeNT was injected into layer 5 of the right visual cortex in a final volume of 1.0 μl at a rate of 100 nl/min (coordinates: 3 mm lateral, 7 mm posterior of bregma, 1.0 mm deep from the pia). An ECoG transmitter (catalog #A3028E, Open Source Instruments) was implanted subcutaneously with a subdural intracranial recording electrode positioned above the injection site. A reference electrode was implanted in the contralateral hemisphere. A cannula (Plastics One) was positioned above the injection site for delivery of lentiviral vectors 11 or 14 d later. Each rat received a maximum of 2.0 μl of lentivirus injected directly into the seizure focus. Animals injected with TeNT were housed separately in Faraday cages for the duration of the study. For the TLE model, status epilepticus (SE) was induced using kainic acid (KA) administered according to a previously described protocol (Hellier et al., 1998). Briefly, adult male Sprague Dawley rats (weight, 200–250 g) were injected intraperitoneally with KA (Tocris Bioscience) dissolved in sterile 0.9% saline (10 mg/ml). Injections were administered hourly at a dose of 5 mg/kg until class III, IV, or V seizures were evoked (scored according to a modified Racine's scale (Racine, 1972; Ben-Ari, 1985)). KA administration was halted when animals reached class V seizures (rearing with forelimb clonus and falling over) or when the total dose of KA reached 45 mg/kg. Animals were included in the study if there was continuous motor seizure activity for 2 h following the final dose of KA. Ten to 12 weeks after the induction of SE, rats were implanted with ECoG transmitters and bilateral guide cannulae for access to the dorsal and ventral hippocampus. After 4 weeks of baseline ECoG recording, animals were injected via the guide cannulae with a total of 8.0 μl of either the AAV-CaMKII-EKC vector (10× dilution) or its titer-matched AAV-CaMKII-GFP control. Vectors were delivered bilaterally into the dorsal and ventral hippocampus (two injection sites in each hemisphere) using the following coordinates: for dorsal hippocampus, ±2.8 mm lateral, 3.2 mm posterior of bregma, 3.1 mm deep from the pia; and for ventral hippocampus, ±4.2 mm lateral, 5.2 mm posterior of bregma, 4.5 mm deep from the pia. Injections were administered at a rate of 200 nl/min, and the needle was left in place for 5 min after each injection.

##### ECoG acquisition and analysis.

ECoG was recorded continuously for up to 6 weeks in the Lenti-CMV-*KCNA1* pilot study and Lenti-CaMKII-EKC trial, and 13 weeks in the AAV-CaMKII-EKC trial. Data were acquired using A3028E implantable transmitters (0.3–160 Hz, 512 samples/s) and ancillary receivers and software (Open Source Instruments). For the Lenti-CMV-*KCNA1* pilot study and Lenti-CaMKII-EKC trial, spontaneous seizures were detected from chronic recordings as previously described (Chang et al., 2018; Lieb et al., 2018). For the AAV-CaMKII-EKC trial, seizure detection was performed using semiautomated supervised learning (https://github.com/jcornford/pyecog). Briefly, a library of seizures was first created from events validated by visual inspection of ECoG traces (and video recordings where available). Library seizures were then divided into short epochs of 5 s and random forest discriminative classification models trained to distinguish between epochs extracted from seizure or baseline periods. To facilitate classification, the discriminative models were trained using features extracted from each epoch, such as power in frequency bands and line-length. Discriminative model predictions of consecutive epochs were combined by treating the predictions as a sequence of observations generated by a hidden Markov model and applying the forward–backward algorithm. To parameterize the hidden Markov model, emission probabilities were calculated from classifier predictions, and manual annotations were treated as hidden states. In all trials, recordings algorithmically classified as seizure activity were verified by visual inspection.

##### Immunohistochemistry.

One week after injection of the Lenti-CaMKII-EKC vector, rats were terminally anesthetized with sodium pentobarbital (Euthatal, Merial) and transcardially perfused with cold (4°C) heparinized PBS (80 mg/L heparin sodium salt; Sigma-Aldrich) followed by 4% paraformaldehyde (PFA) in PBS (Santa Cruz Biotechnology). Brains were removed and postfixed in 4% PFA at 4°C for a further 24 h. After washing in PBS, brains were sliced into 70 μm coronal sections using a vibrating microtome (Leica) and stored free floating at 4°C in PBS plus 0.02% sodium azide (Sigma-Aldrich). For antibody staining, slices were permeabilized for 20 min in PBS plus 0.3% Triton X-100 (Sigma-Aldrich) before blocking for 1 h in PBS plus 0.3% Triton X-100, 1% bovine serum albumin (Sigma-Aldrich) and 4% goat serum (Sigma-Aldrich). Slices were incubated overnight at 4°C in PBS plus 0.3% Triton X-100 and a rabbit anti-NeuN (diluted 1:750; catalog #ab177487, Abcam), mouse anti-GFAP (diluted 1:500; catalog #MAB3402, Merck Millipore), or mouse anti-GAD67 (diluted 1:500; catalog #MAB5406, Merck Millipore) primary antibody. After three 10 min washes in PBS, slices were incubated at room temperature for 3 h in PBS plus the relevant Alexa Fluor 594-conjugated secondary antibody: goat anti-rabbit (catalog #A-11037, Thermo Fisher Scientific) or goat anti-mouse (catalog #A-11005, Thermo Fisher Scientific), both diluted 1:750. After a further three 10 min washes in PBS, slices were mounted onto plain glass microscope slides (Thermo Fisher Scientific) using Vectashield HardSet mounting medium (Vector Laboratories) and borosilicate glass coverslips (VWR). Bright-field and fluorescence images were acquired using one of the following two microscopes: an Axio Imager A1 Fluorescence Microscope (Axiovision LE software), equipped with 2.5×, 10×, and 40× EC Plan-Neofluar nonimmersion objectives; or an inverted LSM 710 confocal laser scanning microscope (ZEN 2009 software) equipped with 40× and 63× EC Plan-Neofluar oil-immersion objectives (all Zeiss). For confocal fluorescence microscopy, destabilized copGFP (dscGFP) and Alexa Fluor 594 were excited with the 488 and 561 nm lines of an argon or diode-pumped solid-state laser (DPSS Lasers), respectively. All image processing was performed using ImageJ software. Composite images were assembled using the MosaicJ ImageJ plugin.

##### Experimental design and statistical analysis.

Data from the Lenti-CMV-*KCNA1* pilot study were used to determine sample sizes for the EKC trials. We estimated that the maximal weekly seizure frequency would double from baseline, and we wished to detect with 80% power a 40% reduction from this maximum at *p* < 0.05. Given a mean baseline weekly seizure frequency of 5 or above, a modification of Lehr's formula (Lehr, 1992) for the Poisson distribution suggested that seven to eight animals per group would be sufficient to detect a reduction in seizure frequency from 10 to 6 per week. Our modified Lehr's formula is given by the following equation:

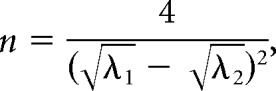
 where *n* is the size of each sample (treatment group), λ_1_ is the mean weekly seizure frequency before treatment, and λ_2_ is the mean weekly seizure frequency after treatment.

**Figure 1.**
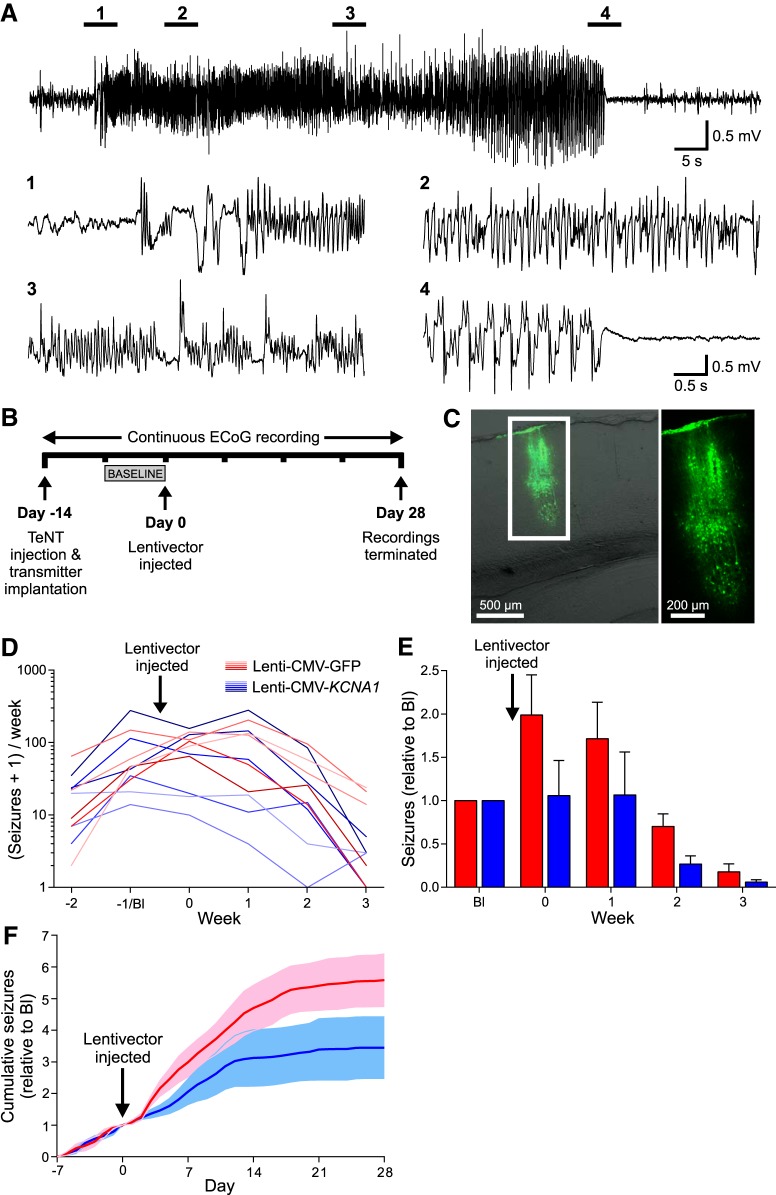
A pilot study suggests that *KCNA1* gene therapy can suppress genuine discrete seizures in the visual cortex TeNT model of FNE. ***A***, Representative occipital lobe seizure experienced by an adult rat 2 weeks after the injection of TeNT into the primary visual cortex. Expanded sections are taken at the times indicated. ***B***, Timeline highlighting key experimental milestones. ***C***, Neuronal transduction with the Lenti-CMV-*KCNA1* vector was restricted to a narrow column of cortex surrounding the site of injection. ***D***, Number of seizures (per week) experienced by animals injected with the Lenti-CMV-*KCNA1* vector (blue; *n* = 6) or its GFP-only Lenti-CMV-GFP control (red; *n* = 5). Data are plotted on a logarithmic scale after incrementing each seizure count by 1 to avoid zero values. ***E***, Normalized seizure frequency (per week) for the two groups. The numbers of seizures experienced each week were normalized to the number experienced by each animal in the week preceding treatment (week Bl). ***F***, Normalized cumulative seizure frequency (per day). Cumulative seizure counts were also normalized to the total number experienced in week Bl. Data in ***E*** and ***F*** are presented as the mean ± SEM.

**Figure 2.**
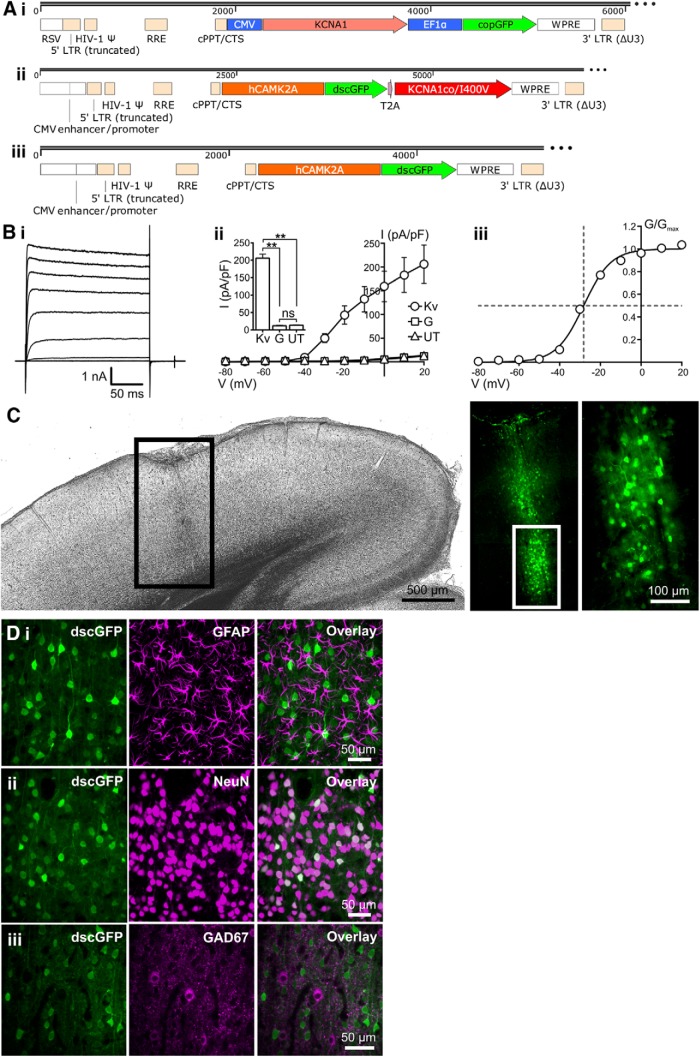
Design and characterization of an EKC gene therapy optimized for clinical translation. ***A***, Transfer plasmid maps for the Lenti-CMV-*KCNA1* pilot vector (***i***), the optimized Lenti-CaMKII-EKC vector (***ii***), and its Lenti-CaMKII-GFP control (***iii***). RSV, Rous sarcoma virus promoter; LTR, long terminal repeat; HIV-1 Ψ, HIV-1 packaging signal; RRE, Rev response element; cPPT/CTS, central polypurine tract and central termination sequence; EF1α, elongation factor 1 α promoter; WPRE, woodchuck hepatitis virus post-transcriptional regulatory element. ***B***, Heterologous expression of functional Kv1.1 channels from the optimized Lenti-CaMKII-EKC transfer plasmid. ***i***, Representative current–time trace from a Neuro-2a cell transfected with the Lenti-CaMKII-EKC transfer plasmid. ***ii***, Plot of mean current density against voltage for cells transfected with the Lenti-CaMKII-EKC transfer plasmid (Kv; *n* = 13), cells transfected with the Lenti-CaMKII-GFP control plasmid (G; *n* = 8), and untransfected controls (UT; *n* = 10). Inset, Histogram showing differences in current density among the three groups during the voltage step to +20 mV (Kv vs UT, *p* = 0.0013; Kv vs G, *p* = 0.0012; UT vs G, *p* = 0.82; ns = not significant; Welch's one-way ANOVA with Games–Howell *post hoc* tests). ***iii***, Plot of mean normalized conductance against voltage for cells transfected with the Lenti-CaMKII-EKC transfer plasmid. Data are fit with a single Boltzmann function. The *V*_0.5_ value of −28.2 mV is similar to values obtained from HEK293 cells transfected with CMV-driven, wild-type *KCNA1* (−32.8 ± 0.9 mV; Tomlinson et al., 2013). All error bars represent the SEM. ***C***, Bright-field and fluorescence images of a brain slice from a rat injected in the left visual cortex with 1.25 μl (∼3.0 × 10^6^ IU) of the Lenti-CaMKII-EKC vector. The pattern of transduction is similar to that observed with the Lenti-CMV-*KCNA1* vector. ***D***, Immunohistochemical assessment of the cell type specificity of EKC expression. ***i***, There was no overlap between transduced neurons expressing dscGFP and astrocytes stained for GFAP. ***ii***, There was 100% overlap between dscGFP^+^ cells and neurons stained for NeuN. ***iii***, Minimal overlap was observed between dscGFP^+^ cells and inhibitory interneurons stained for GAD67. **p < 0.002.

**Figure 3.**
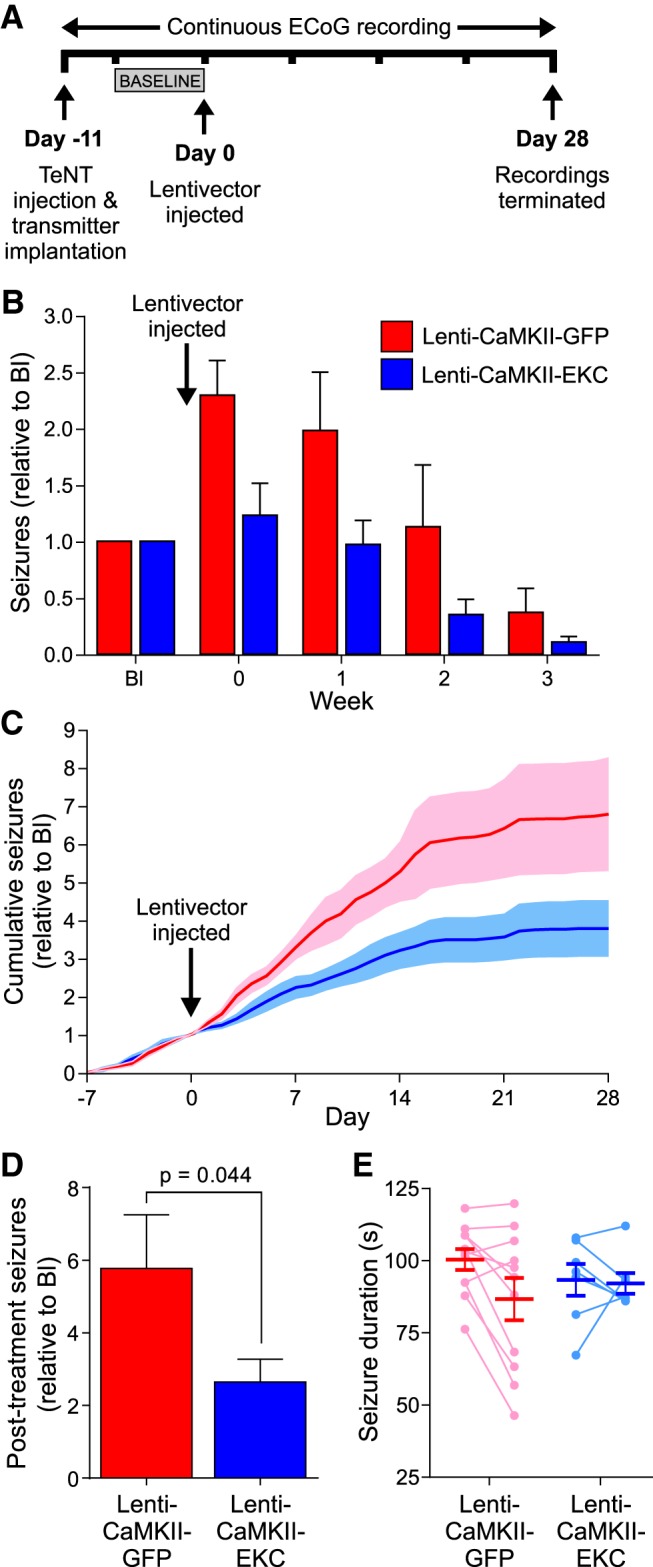
EKC gene therapy robustly reduces seizure frequency in a blinded, randomized, placebo-controlled preclinical trial. ***A***, Timeline highlighting key experimental milestones. Note the injection of lentiviral vectors 11 rather than 14 d after TeNT delivery. ***B***, Normalized seizure frequency (per week) for animals treated with the Lenti-CaMKII-EKC lentivector (blue; *n* = 7/6) or its Lenti-CaMKII-GFP control (red; *n* = 11). ***C***, Normalized cumulative seizure frequency (per day). ***D***, Normalized post-treatment seizure totals. ***E***, Individual and overall average seizure durations before (left) and after (right) treatment. Data in ***B–E*** are presented as the mean ± SEM.

**Figure 4.**
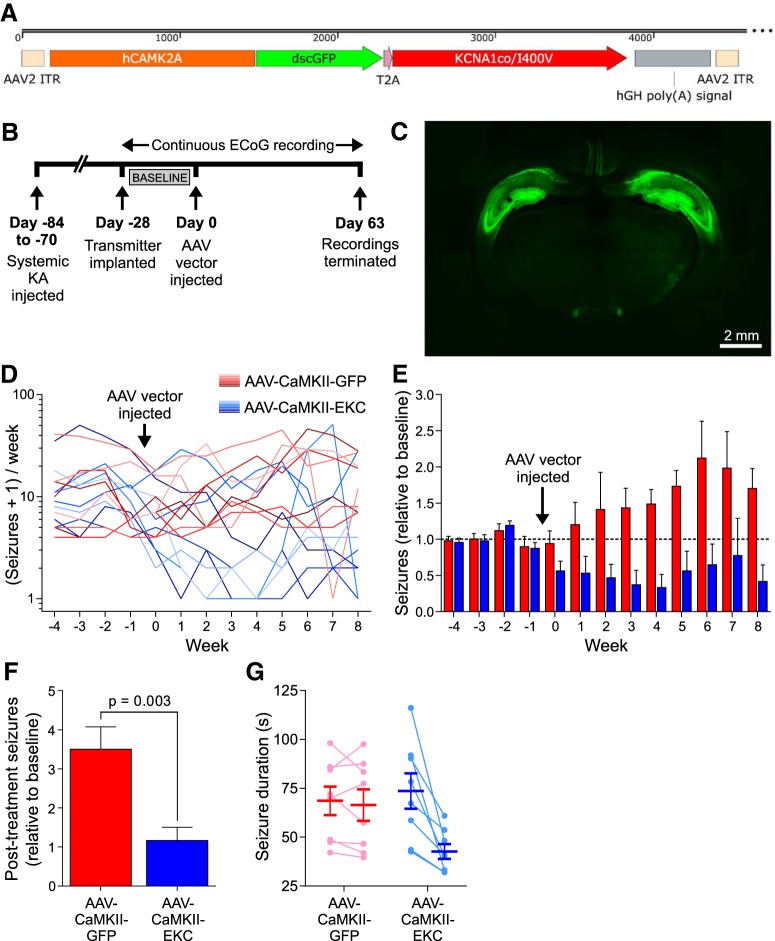
EKC gene therapy is effective in a model of TLE. ***A***, Transfer plasmid map for the AAV-CaMKII-EKC vector. hGH poly(***A***) signal, Human growth hormone polyadenylation signal. ***B***, Timeline highlighting key experimental milestones. ***C***, Representative fluorescence image of a brain slice from a rat injected in the bilateral hippocampus with 8.0 μl of undiluted AAV-CaMKII-EKC vector. ***D***, Number of seizures (per week) experienced by animals injected with the AAV-CaMKII-EKC vector (blue; *n* = 8) or its AAV-CaMKII-GFP control (red; *n* = 8). Data are plotted on a logarithmic scale after incrementing each seizure count by 1 to avoid zero values. ***E***, Normalized seizure frequency (per week) for the two groups. ***F***, Normalized post-treatment seizure totals. ***G***, Individual and overall average seizure durations before (left) and after (right) treatment. Data in ***E–G*** are presented as the mean ± SEM.

Seizure counts in the baseline periods preceding treatment were compared using a two-tailed Mann–Whitney *U* test. The effects of treatment on normalized seizure frequency (Fig. 1*E*, Figs. 3*B*, Fig. 4*E*) were analyzed using a generalized log-linear mixed model with random effect of animal (autoregressive covariance) and fixed effects of treatment group, week, and the interaction between treatment group and week. The effects of treatment on overall seizure burden (see Figs. 3*D*, Fig. 4*F*) and seizure clustering were analyzed using a two-tailed Mann–Whitney *U* test for the Lenti-CaMKII-EKC trial, and a two-tailed independent-samples *t* test for the AAV-CaMKII-EKC trial. The effects of treatment on seizure duration (see Figs. 3*E*, Fig. 4*G*) were analyzed using a two-way repeated-measured ANOVA with factors of time point (pretreatment or post-treatment) and treatment group. Current densities at +20 mV (Fig. 2*Bii*) were compared using a Welch's one-way ANOVA followed by Games–Howell *post hoc* tests.

## Results

### A pilot study shows that *KCNA1* gene therapy suppresses spontaneous seizures in a visual cortex epilepsy model

We first asked whether the CMV-driven *KCNA1* lentivector (Lenti-CMV-*KCNA1*) used previously in a model of EPC (Wykes et al., 2012) was also effective in a neocortical epilepsy model characterized by discrete seizures (Fig. 1*A*). Epilepsy was induced in adult rats with a single injection of TeNT into the primary visual cortex. Seizures in this model typically last between 50 and 200 s; are accompanied by unilateral, bilateral, or generalized convulsions; and evolve over several weeks before fading (Chang et al., 2018; Lieb et al., 2018). To monitor local electrographic activity, a wireless ECoG transmitter was implanted with a subdural intracranial recording electrode positioned above the injection site. Two weeks after TeNT administration, following the establishment of epilepsy, animals were randomized into two groups and injected via a preimplanted cannula with either the Lenti-CMV-*KCNA1* vector or a Lenti-CMV-GFP control vector expressing only GFP. Injections were delivered directly into the seizure focus and followed by a further 4 weeks of ECoG recording (Fig. 1*B*).

The Lenti-CMV-*KCNA1* lentivector transduced neurons within a narrow column of the cortex (Fig. 1*C*). As is typical of this model (Chang et al., 2018; Lieb et al., 2018), the total number of seizures experienced by each animal over the 6 weeks of recording was highly variable (Fig. 1*D*). Consequently, to compare seizure frequency between the two treatment groups the numbers of seizures experienced each week were normalized to the number experienced in the week preceding treatment (week −1 or Bl week). Despite the small sample size (six treated vs five controls), the Lenti-CMV-*KCNA1* vector significantly reduced normalized seizure frequency compared with controls in the weeks following treatment (generalized log-linear mixed model on weeks 0–3, treatment group × week interaction effect: *F*_(1,40)_ = 4.851, *p* = 0.033; Fig. 1*E*). The therapeutic effect emerged rapidly; plots of normalized cumulative daily seizure frequency for the two groups diverged within 3 d of lentivector injection, consistent with rapid transgene expression (Fig. 1*F*).

This pilot study strongly suggests that *KCNA1* gene therapy can suppress spontaneous discrete neocortical seizures. However, the Lenti-CMV-*KCNA1* vector tested is poorly suited for clinical translation. We therefore set out to develop an optimized lentivector with improved safety and efficacy.

### Design and characterization of a Lenti-CaMKII-EKC gene therapy optimized for clinical translation

The transfer plasmid used to synthesize the optimized lentivector differed from the original Lenti-CMV-*KCNA1* construct in several ways (Fig. 2*A*). The non-cell-type specific CMV promoter was replaced with a 1.3 kb human *CAMK2A* promoter to bias expression to excitatory neurons (Dittgen et al., 2004; Yaguchi et al., 2013). The *KCNA1* gene was codon optimized for expression in human cells, and mutated to introduce an I400V amino acid substitution normally generated by RNA editing. This substitution elicits a 20-fold increase in the rate at which Kv1.1 channels recover from inactivation (Bhalla et al., 2004). For preclinical evaluation, the coding sequence of a short-lived dscGFP reporter was linked to the EKC gene by a T2A element, which permits dual peptide expression from a single promoter. To ensure that the EKC construct could produce functional Kv1.1 channels, we performed whole-cell patch-clamp recordings in transfected Neuro-2a cells, a line selected for its high *Camk2a* promoter activity. Robust non-inactivating Kv1.1 currents were recorded in cells transfected with the EKC plasmid (Fig. 2*B*).

The EKC transfer plasmid was packaged into a nonintegrating lentiviral vector (Yáñez-Muñoz et al., 2006; Rahim et al., 2009). When injected into the rat visual cortex, this Lenti-CaMKII-EKC vector drove strong, localized expression of the dscGFP reporter (Fig. 2*C*). Imaging of sequential brain slices yielded an estimated transduction volume of ∼0.074 mm^3^ (data not shown). Immunohistochemistry revealed no visible overlap between dscGFP expression and glial fibrillary acidic protein (GFAP) staining (0 of 512 dscGFP^+^ cells stained for GFAP, *n* = 3 animals; Fig. 2*Di*). In contrast, all dscGFP^+^ cells stained positively for the neuronal marker NeuN (714 of 714 cells, *n* = 3 animals; Fig. 2*Dii*). These data indicate that transgene expression from the EKC lentivector is restricted to neurons. There was minimal overlap between dscGFP expression and staining for glutamic acid decarboxylase 67 (GAD67), an enzymatic marker for GABAergic neurons (3 of 603 dscGFP^+^ cells stained for GAD67, *n* = 3 animals; Fig. 2*Diii*). This suggests that EKC transgene expression is largely restricted to excitatory neurons.

### Lenti-CaMKII-EKC gene therapy reduces seizure frequency in a blinded, randomized preclinical trial

To test the therapeutic efficacy of the Lenti-CaMKII-EKC vector, we designed a blinded, randomized, placebo-controlled preclinical trial, and selected normalized seizure frequency as the primary outcome measure. Eleven days after the injection of TeNT into the visual cortex, 26 rats were randomized into two groups and injected via a preimplanted cannula with either the Lenti-CaMKII-EKC vector or its dscGFP-only Lenti-CaMKII-GFP control. ECoG recordings were continued for a further 4 weeks. The timeline was altered from that of the pilot study to treat after 11 d to capture the period when seizure activity is at its highest (2–4 weeks following TeNT injection; Fig. 3*A*).

To minimize the confounding influence of animals that displayed a very low seizure frequency before treatment and were therefore unlikely to develop chronic seizures, subjects were excluded if they exhibited fewer than five seizures in the week preceding lentiviral delivery (the baseline week). This criterion, established before commencement of the final preclinical trial and applied before unblinding, led to the exclusion of eight animals (six EKC, two control). Of the remaining 18 animals, all but one survived for the duration of recording. This rat (from the EKC group) was culled in the final week due to detachment of its headpiece. However, because the subject had already passed through the period of peak seizure activity, and to maximize the data obtained from the study, this incomplete dataset was included in the overall analysis. Again, this decision was made before unblinding.

There was no significant difference between the treatment groups in the number of seizures experienced in the baseline week [Lenti-CaMKII-GFP: median, 11; interquartile range (IQR), 10–26; Lenti-CaMKII-EKC: median, 10; IQR, 7.5–12; Mann–Whitney *U* test, *p* = 0.185]. Analysis of the primary outcome measure indicated that Lenti-CaMKII-EKC therapy robustly decreased normalized seizure frequency compared with controls in the weeks following treatment (generalized log-linear mixed model on weeks 0–3; treatment group × week interaction effect: *F*_(1,67)_ = 40.137, *p* < 0.001; Fig. 3*B*). The size of the effect was larger than that observed in the pilot study, suggesting that the EKC gene is more effective than its wild-type *KCNA1* counterpart at suppressing neuronal hyperexcitability. As in the pilot study, the reduction in seizure frequency lasted for the duration of recording, and the absolute effect size only decreased as seizures abated in the control group. Again, the therapeutic effect emerged rapidly, with plots of normalized cumulative daily seizure frequency for the two groups diverging 2 d after treatment (Fig. 3*C*). To determine the effect of Lenti-CaMKII-EKC therapy on overall seizure burden, which is an important determinant of comorbidities and mortality in epilepsy (Trinka et al., 2013), we compared total post-treatment seizure counts (normalized to baseline) between the two treatment groups. Lenti-CaMKII-EKC therapy significantly reduced the overall seizure burden (Mann–Whitney *U* test, *p* = 0.044; Fig. 3*D*). Lenti-CaMKII-EKC therapy had no significant effect on the duration of seizures that persisted after treatment (two-way repeated-measures ANOVA on average durations before and after treatment; treatment group × time point interaction effect: *F*_(1,16)_ = 2.640, *p* = 0.124; Fig. 3*E*).

The visual cortex TeNT model of FNE exhibits pronounced seizure clustering (Chang et al., 2018; Lieb et al., 2018), which is also common in human epilepsies (Karoly et al., 2016) and has been reported to correlate with poor clinical outcome and quality of life, and even mortality (Sillanpää and Schmidt, 2008). We therefore asked whether Lenti-CaMKII-EKC therapy influenced seizure clustering in the current study. The degree of clustering was quantified by calculating the post-treatment Fano factor—the ratio of the mean number of seizures to the variance—for control and EKC-treated animals. This revealed a nonsignificant trend for a lower degree of clustering, as indicated by the Fano factor, in EKC-treated animals compared with controls (Lenti-CaMKII-GFP: median, 4.58; IQR, 1.91–14.57; Lenti-CaMKII-EKC: median, 2.06; IQR, 0.86–3.25; Mann–Whitney *U* test, *p* = 0.073).

### AAV-CaMKII-EKC gene therapy suppresses seizures in a temporal lobe epilepsy model

To determine whether the efficacy of EKC gene therapy was specific to lentiviral treatment of FNE, we performed an additional randomized, blinded trial in a model of TLE induced by systemic KA injection. Because the seizure focus is more diffuse in this model, we delivered the EKC gene (again under the *CAMK2A* promoter) bilaterally to the hippocampi using a rAAV2/9 vector. Rats were implanted with wireless ECoG transmitters 10–12 weeks after the induction of SE by intraperitoneal KA. After recording baseline seizure activity for 4 weeks, 16 epileptic animals were randomized into two groups for injection via preimplanted cannulae with either an AAV-CaMKII-EKC vector (Fig. 4*A*) or a dscGFP-only AAV-CaMKII-GFP control. ECoG recordings were continued for a further 9 weeks (Fig. 4*B*).

The AAV-CaMKII-EKC vector drove strong, widespread expression of the dscGFP reporter throughout the hippocampus (Fig. 4*C*). As for the visual cortex model of FNE, seizure counts in the TLE model were highly variable (Fig. 4*D*), so comparisons between the two treatment groups were made after normalizing weekly counts to the mean number of seizures experienced per week in the baseline period. The analysis was performed blind to treatment. There was no significant difference between the groups in the number of seizures experienced during this baseline period (AAV-CaMKII-GFP: median, 34; IQR, 15–62.75; AAV-CaMKII-EKC: median, 41.5; IQR, 22.25–55.5; Mann–Whitney *U* test, *p* = 0.521). AAV-CaMKII-EKC therapy robustly decreased normalized seizure frequency compared with controls in the weeks following treatment (generalized log-linear mixed model on weeks 0–8; treatment group × week interaction effect: *F*_(1,126)_ = 6.331, *p* = 0.013; Fig. 4*E*). AAV-CaMKII-EKC therapy also led to a significant reduction in the overall seizure burden (independent-samples *t* test, *t*_(14)_ = 3.54, *p* = 0.003; Fig. 4*F*). In contrast to the visual cortex TeNT study above, the average seizure duration decreased after treatment (two-way repeated-measures ANOVA on average durations before and after treatment; treatment group × time point interaction effect: *F*_(1,14)_ = 11.20, *p* = 0.005; Fig. 4*G*). Seizure clustering was unaffected (independent-samples *t* test comparing post-treatment Fano factors, *t*_(14)_ = 1.11, *p* = 0.285).

## Discussion

The present study shows EKC gene therapy to be effective in models of both FNE and TLE, providing strong justification for further clinical development.

Early studies of gene therapy for epilepsy focused on acutely precipitated seizures, which often translate poorly (Galanopoulou et al., 2012). More recent strategies, mainly involving AAVs in models of TLE, have shown that the development of seizures after an epileptogenic insult (epileptogenesis) can be attenuated (Haberman et al., 2003; Richichi et al., 2004; Lin et al., 2006; McCown, 2006; Kanter-Schlifke et al., 2007; Noè et al., 2008; Bovolenta et al., 2010; Woldbye et al., 2010; Nikitidou et al., 2014). Here we show, in models of both TLE and FNE using both AAV and lentiviral vectors, that potassium channel gene therapy can suppress spontaneous recurrent seizure activity that is already established.

We have previously shown that the overexpression of Kv1.1 can reduce the frequency of brief (<1 s), high-frequency epileptiform discharges in a motor cortex TeNT model of EPC (Wykes et al., 2012). However, that study did not investigate whether Kv1.1 overexpression could inhibit discrete seizures lasting 1–2 min, which are more typical of common forms of focal epilepsy. We show here, in three independent trials, that Kv1.1 overexpression is indeed sufficient to reduce the frequency of discrete, long-lasting seizures. *In vitro* studies have demonstrated that Kv1.1 overexpression reduces both intrinsic neuronal excitability and glutamate release from transduced pyramidal neurons (Heeroma et al., 2009; Wykes et al., 2012), which may provide a mechanism for limiting seizure initiation. Importantly, both these effects on neuronal properties are graded, with neither neuronal excitability nor neurotransmitter release completely abolished.

Interestingly, AAV-CaMKII-EKC therapy in the TLE model reduced both the frequency and the duration of seizures, while Lenti-CaMKII-EKC therapy in the FNE model reduced only seizure frequency. This difference may be explained by the spread of AAV and lentiviral vectors through the brain parenchyma in relation to seizure-generating networks. In the FNE model, the motor convulsions that accompany the majority of seizures suggest that seizure initiation is rapidly followed by propagation to brain areas outside the TeNT-injected primary focus (Chang et al., 2018; Lieb et al., 2018). Because Lenti-CaMKII-EKC remained confined to the injection site, such propagation would leave EKC channels unable to influence the termination and, thus, the duration of seizure activity. Conversely, the fact that bilateral hippocampal AAV-CaMKII-EKC treatment reduces seizure duration in the TLE model suggests that activity in limbic structures contributes to determine the evolution of individual seizures.

Lentiviral gene therapy approaches are becoming more common in CNS disorders, and have shown good safety and tolerability even in extended trials (Palfi et al., 2014). However, a potential safety concern with retroviral vectors is the inherent risk of insertional mutagenesis (Hacein-Bey-Abina et al., 2003; Baum et al., 2004). This risk can be minimized by rendering vectors integration deficient. The popularity of nonintegrating lentiviruses for therapeutic gene transfer is growing, and the vectors have already demonstrated preclinical efficacy in the treatment of degenerative retinal disease and hemophilia B (Yáñez-Muñoz et al., 2006; Suwanmanee et al., 2014). The nonintegrating EKC lentivirus described here drove strong, localized transgene expression after direct injection into the rat neocortex, and rapidly and persistently suppressed focal seizure activity. This supports the use of integration-deficient vectors as safe, effective delivery tools for gene therapy of neurological disease.

In the case of epilepsy, an additional safety concern is the possibility of potassium channel overexpression in interneurons, which could aggravate seizure activity by exacerbating rather than attenuating local excitability. To mitigate this risk, we have used a human *CAMK2A* promoter that in rats led to very little expression in GABAergic cells. Promoter specificity can differ between species (Yaguchi et al., 2013; Lerchner et al., 2014), and the specificity of the human *CAMK2A* promoter for excitatory glutamatergic neurons will ultimately need to be validated in the human brain. Evidently, if EKC gene therapy is to progress to the clinic, such validation will need to be performed in the absence of a fluorescent reporter.

Because the role of potassium channels, including Kv1.1, in regulating neuronal excitability is conserved across a broad range of neurons, potassium channel overexpression may hold therapeutic promise in the treatment of other diseases characterized by neuronal hyperexcitability. There is currently an unmet clinical need for new treatments of chronic pain, and a variety of gene therapy approaches aimed at reducing the excitability of dorsal root ganglion neurons has already demonstrated preclinical efficacy (Snowball and Schorge, 2015). Other disorders such as Parkinson's disease are associated with excessive activity in specific groups of neurons (Lobb, 2014) and could be candidates for treatment with an appropriate combination of potassium channel subtype and cell type-specific promoter.
